# Supplementary material for: Mesenchymal Stromal Cells for the Enhancement of Surgical Flexor Tendon Repair in Animal Models: A Systematic Review and Meta-Analysis
Source: Bioengineering (Basel). 2024 Jun 27;11(7):656. doi: 10.3390/bioengineering11070656 (PMC11274147; doi:10.3390/bioengineering11070656)
Supplement: Supplementary file 1 [file bioengineering-11-00656-s001.zip › Analytic code.pdf]

Analytic code for meta-analyses and subgroup analyses

For proportions (e.g. complication rate):

```
m.prop <- metaprop(event = Event,
              n = N,
              studlab = Author,
              data = Superficial_Infection,
              method = "Inverse",
              MH.exact = TRUE,
              incr = 0,
              sm = "PLOGIT",
              fixed = FALSE,
              random = TRUE,
              hakn = TRUE,
              method.tau = "PM",
              prediction = TRUE)
```

For continuous data (calculating SMD):

```
m.cont <- metacont(n.e = n.e,
              mean.e = mean.e,
              sd.e = sd.e,
              n.c = n.c,
              mean.c = mean.c,
              sd.c = sd.c,
              studlab = author,
              data = OMAS_Difference_Regression,
              sm = "SMD",
              method.smd = "Cohen",
              fixed = FALSE,
              random = TRUE,
              method.tau = "SJ",
              prediction = TRUE,
              hakn = TRUE)
```

For subgroup analysis:

```
update.meta(m.cont,
        subgroup = RoB,
        tau.common = FALSE)
```
