# Supplementary material for: Mesenchymal Stromal Cells for the Enhancement of Surgical Flexor Tendon Repair in Animal Models: A Systematic Review and Meta-Analysis
Source: Bioengineering (Basel). 2024 Jun 27;11(7):656. doi: 10.3390/bioengineering11070656 (PMC11274147; doi:10.3390/bioengineering11070656)
Supplement: Supplementary file 1 [file bioengineering-11-00656-s001.zip › Supplementary Figure 1.pdf]

Supplementary Figure 1. Contour-enhanced funnel plot for publication bias

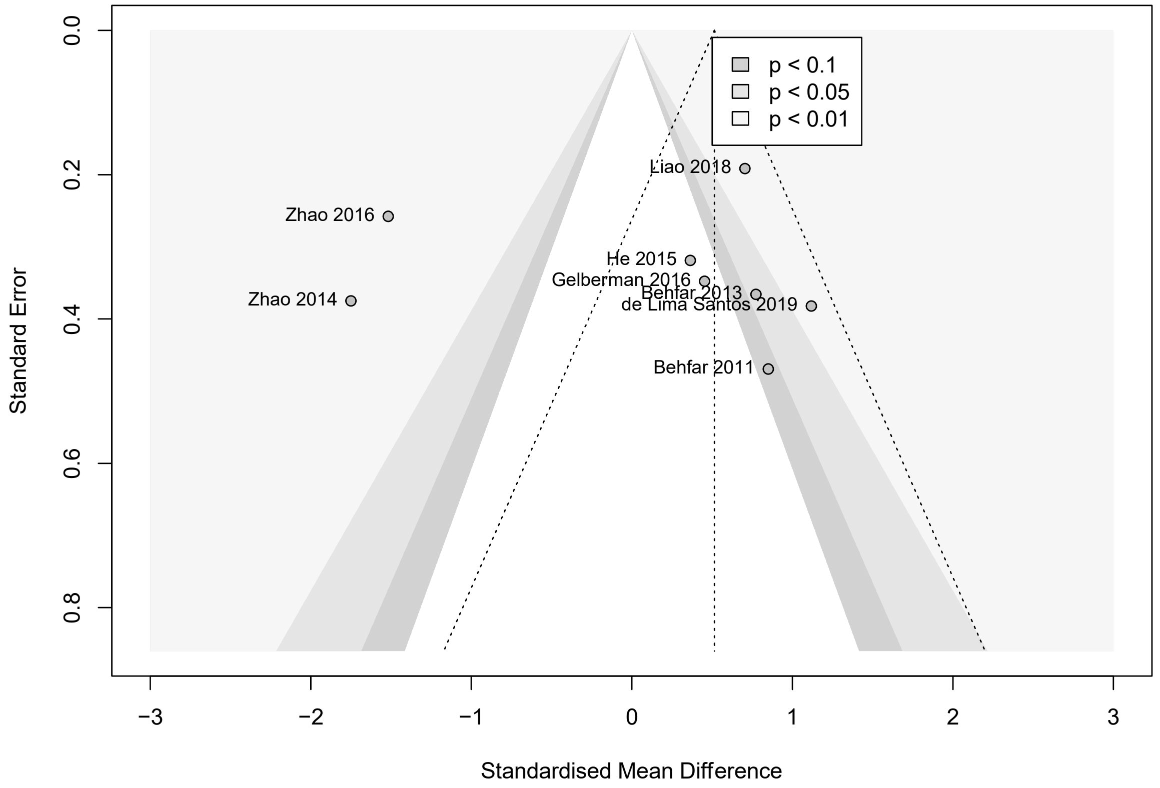

Egger's test of the intercept for publication bias: $p = 0.449$.

Egger's test does not indicate the presence of funnel plot asymmetry.
