# Supplementary material for: Mesenchymal Stromal Cells for the Enhancement of Surgical Flexor Tendon Repair in Animal Models: A Systematic Review and Meta-Analysis
Source: Bioengineering (Basel). 2024 Jun 27;11(7):656. doi: 10.3390/bioengineering11070656 (PMC11274147; doi:10.3390/bioengineering11070656)
Supplement: Supplementary file 1 [file bioengineering-11-00656-s001.zip › Supplementary Table 1.pdf]

**Supplementary table 1:** Detailed search strategy

| Tendon | MSC variations | Injury variations | Final |
|---|---|---|---|
| 1) Tendon | 2) Mesenchymal stem cell<br>3) Mesenchymal stromal cell<br>4) MSC<br>5) (BM or bone-marrow or bone marrow) derived<br>6) Adipose-derived or adipose derived or ASC<br>7) (Blood or peripheral blood or peripheral-blood or PB) derived<br>8) 2 OR 3 OR 4 OR 5 OR 6 OR 7 | 9) Ruptur*<br>10) Tear<br>11) Lacerat*<br>12) Injur*<br>13) 8 OR 9 OR 10 OR 11 | 14) 1 AND 8 AND 13 |
